# Histological grading in primary membranous nephropathy is essential for clinical management and predicts outcome of patients

**DOI:** 10.1111/his.13955

**Published:** 2019-10-03

**Authors:** Maria J Stangou, Smaragdi Marinaki, Evangelos Papachristou, George Liapis, Panagiotis Pateinakis, Hara Gakiopoulou, Christina Nikolaidou, Kyriaki Kolovou, Ioanna‐Theologia Lampropoulou, Synodi Zerbala, Panagiota Papadea, Evangelia Dounousi, Olga Balafa, Paraskevi Pavlakou, Aimilios Andrikos, Eufemia Balassi, Panagiota Manolakaki, George Moustakas, Dimitra Galitsiou, Efstathios Mitsopoulos, Christina Vourlakou, Vasiliki Choulitoudi, Paraskevi‐Evi Andronikidi, Ioannis Stefanidis, Spyridon Golfinopoulos, Eugene Dafnis, Kostas Stylianou, Stylianos Panagoutsos, Apostolos Papadogianakis, Ioannis Tzanakis, Athanasios Sioulis, Demetrios Vlahakos, Irene Grapsa, Maria Tsilivigkou, Nikolaos Kaperonis, Christos Paliouras, Christos Dioudis, Sophia Spaia, Theofanis Apostolou, Christos Iatrou, John Boletis, Dimitrios Goumenos, Aikaterini Papagianni

**Affiliations:** ^1^ Hippokration General Hospital Aristotle University Thessaloniki Greece; ^2^ National and Kapodistrian University Laiko General Hospital Athens Greece; ^3^ University Hospital of Patras Patras Greece; ^4^ Papageorgiou General Hospital of Thessaloniki Thessaloniki Greece; ^5^ General Hospital of Nikaia Piraeus Greece; ^6^ University Hospital of Ioannina Ioannina Greece; ^7^ Hatzikosta General Hospital of Ioannina Ioannina Greece; ^8^ Gennimatas General Hospital of Athens Athens Greece; ^9^ Evaggelismos General Hospital Athens Greece; ^10^ University Hospital of Larissa Larissa Greece; ^11^ University Hospital of Heraklion Heraklion Crete Greece; ^12^ University Hospital of Alexandroupolis Alexandroupoli Greece; ^13^ Venizelio General Hospital of Heraklion Heraklion Crete Greece; ^14^ General Hospital of Chania Chania Crete Greece; ^15^ AHEPA University General Hospital Thessaloniki Greece; ^16^ Attikon University Hospital National and Kapodistrian University Athens Greece; ^17^ Aretaieio Hospital National and Kapodistrian University of Athens Athens Greece; ^18^ Tzaneion General Hospital of Piraeus Athens Greece; ^19^ Hellenic Red Cross Hospital Korgialeneio‐Benakeio Athens Greece; ^20^ General Hospital of Rhodes Rhodes Greece; ^21^ General Hospital of Drama Drama Greece; ^22^ General Hospital of Thessaloniki ‘Agios Pavlos’ Thessaloniki Greece

**Keywords:** focal sclerosis, interstitial fibrosis, outcome, primary membranous nephropathy, tubular atrophy, vascular hyalinosis

## Abstract

**Aims:**

Diagnosis of primary membranous nephropathy (PMN) is mainly based on immunofluorescence/immunohistochemistry findings. However, assessment of specific features on optical microscopy can help to estimate the severity of the disease, guide treatment and predict the response. The aim of this study was to identify, classify and grade the precise histological findings in PMN to predict renal function outcome and guide treatment.

**Methods and results:**

Histological parameters, including focal segmental sclerosis (FSGS), tubular atrophy (TA), interstitial fibrosis (IF) and vascular hyalinosis (VH), were re‐evaluated in 752 patients with PMN. Their predictive value was estimated separately, and also in a combination score (FSTIV) graded from 0 to 4. Finally, the impact of histology was assessed in the response to immunosuppressive treatment. Mean age of patients was 53.3 (15–85) years and most presented with nephrotic syndrome. FSGS was present in 32% and VH in 51% of the patients, while TA and IF were graded as stage ≥1 in 52% and 51.4%, respectively. The follow‐up period was 122.3 (112–376) months. FSGS, TA and IF and VH were associated with impaired renal function at diagnosis (*P* = 0.02, *P* < 0.0001, *P* = 0.001 and *P* = 0.02, respectively) and at the end of follow‐up (*P* = 0.004, *P* < 0.0001, *P* < 0.0001 and *P* = 0.04, respectively). In multiple regression and binary logistic analysis, the presence of FSGS and degree of TA were the most significant parameters predicting renal function outcome, defined either by eGFR (end), FSGS (*r* = 0.6, *P* < 0.0001) and TA (*r* = 0.6, *P* < 0.0001), or by the endpoint of >50% eGFR reduction, FSGS (*P* = 0.001) and TA (*P* = 0.02). Also, patients presented with FSGS, IF, VH and/or with FSTIV > 1 could benefit from immunosuppression, regardless of clinical presentation.

**Conclusions:**

The presence and degree of four histological indices, FSGS, VH, TA and IF, assessed separately or in combination, and FSTIV score not only predict renal function outcome after long‐term follow‐up, but can also help in the choice of appropriate treatment. Decisions concerning immunosuppressive treatment can be guided by pathology regardless of clinical findings.

## Introduction

Primary membranous nephropathy (PMN) remains the most common cause of nephrotic syndrome in white adults, representing 20–30% of cases in 50–60‐year‐old patients, rising to 40% in ages greater than 60 years.^1^, ^2^, ^3^

Diagnosis of MN is based on optical microscopy and immunohistochemistry and/or immunofluoresence findings, showing thickening of glomerular basement membrane with or without spikes and granular deposition of immunoglobulin (Ig)G and C3 along the glomerular capillary walls. Electron microscopy demonstrates the subepithelial deposits and helps in disease classification.^2^, ^4^, ^5^, ^6^, ^7^ Recent discovery of circulating pathogenic antibodies, the M‐type phospholipase A2 receptor 1 (PLA2R) and the thrombospondin type 1 domain‐containing 7A (THSD7A), supported the theory of immunological pathogenesis of the disease and helped to change the term ‘idiopathic’ MN to ‘primary’ MN (PMN).^8^, ^9^, ^10^, ^11^, ^12^, ^13^ Although the description of PLA2R and THSD7A antibodies has regenerated the clinicians’ wish to substitute a serum or urinary biomarker for renal biopsy, we are far from coming to this point, as renal biopsy gives much more information than a histopathologically unsupported diagnosis. Therefore, renal biopsy remains vital for diagnosis of PMN and assessment of disease severity. Classification of the disease on a scale of 0–IV, based on electron microscopy findings, was an attempt to estimate disease severity and categorise patients according to renal lesions, although it has not always been proved to be of clinical significance.^14^ Also, electron microscopy is not broadly available within pathology centres. Therefore, there is a need to evaluate optical microscopy and define specific features which can be easily assessed in clinical everyday practice, and have the capacity to estimate severity of the disease and guide treatment.

The present study included patients from the Greek Registry of Primary Membranous Nephropathy, part of the Greek Network of Glomerular Diseases. All patients had undergone a thorough investigation to exclude secondary cases; they had a long‐term follow‐up, reaching more than 10 years, and their histological reports were re‐evaluated in an attempt to define parameters which may determine disease progression. Furthermore, we defined specific histological findings which, in combination with clinical symptoms at presentation, may identify the profile of patients who are likely to benefit from immunosuppressive treatment.

## Patients and methods

### Clinical Data, Treatment and Outcome

The clinical data from 752 patients with PMN from 22 centres diagnosed during the period 1995–2015 were obtained from the Registry of Primary Membranous Nephropathy, Greek Network of Glomerular Diseases. Diagnosis was based on optical microscopy and immunohistochemistry and/or immunofluorescence. Electron microscopy was performed on a limited number of patients. Extensive research had excluded secondary cases of MN, such as tumours, systemic diseases, viral infections and previous medication. Renal function and degree of proteinuria at time of diagnosis and at the end of follow‐up, the presence of nephrotic syndrome (NS) and renal function impairment at diagnosis, immunosuppressive treatment and outcome were recorded for all patients.

Patients were treated according to Kidney Disease: Improving Global Outcomes (KDIGO) guidelines, immunosuppressive treatment was added either after a 6‐month trial with RAASi in cases with partial remission or no response, or earlier, in patients with rapidly progressive disease and/or life‐threating complications.

All patients had at least 12 months of follow‐up, unless they reached end‐stage renal disease (ESRD) or died. End of follow‐up was considered the last visit to outpatient clinic, the day started on haemodialysis or death.

The study was approved by the Institutional Review Board of Hippokration General Hospital, Thessaloniki, Greece, Approval number 10/18, approval date 22 March 2016.

### Pathology Review

Renal biopsy reports were re‐evaluated, and characteristics estimated included the presence or absence of focal segmental sclerosis (FSGS), vascular hyalinosis (VH) and immunoglobulin (Ig)G and C3 staining on immunofluoresence and/or immunohistochemistry. The severity of tubular atrophy (TA) and interstitial fibrosis (IF) was rated on a scale of 0, 1 or 2 based on the percentage of affected tubules (<25, 25–50, >50%) or the extension of IF (absent–mild, moderate, severe), respectively.

Combining the four histological parameters, including FSGS (0–1), TA (0–1, 2), IF (0–1, 2) and VH (0–1), we set a FSTIV score, graded in scale from 0 to 4 (when none of the above indices were evident, to one, two, three or four parameters evident, respectively). For practical reasons, each of the histology indices were considered as dichotomous (0 or 1 for present or absent, respectively), TA and IF graded as stage 0 were considered as absent and stages 1 or 2 were considered as present.

### Definitions and Outcome of Renal Function

Patients who presented with eGFR < 60 ml/min/1.73 m^2^, which remained at these levels for at least 3 months, were considered as having chronic kidney disease (CKD). End of follow‐up was considered the last visit to outpatient clinic or, for those who reached ESRD or died, the time when they started on the dialysis method or day of death.

Outcome of renal function was assessed by (i) eGFR at the end of follow‐up; eGFR (end); (ii) response to treatment, defined as complete, partial or no response; and (iii) endpoint of a >50% reduction in eGFR and/or ESRD. The rate of renal function alteration was estimated by the equation [eGFR (end)–eGFR (start)]/follow‐up in years and defined as slope‐eGFR.

eGFR was estimated based on the Modification of Diet in Renal Disease study (MDRD) equation. Response to treatment was defined, according to KDIGO, as complete when proteinuria reduced to <0.3 g/24 h with normal levels of serum albumin and normal renal function, partial as the reduction of proteinuria to >50% from baseline, but still remains between 0.3 and 3.5 g/24 h and stable renal function, and no response, when proteinuria levels remained ≥50% from baseline and/or ≥3.5 g/24 h and/or declining renal function.^15^

### Statistical Analysis

Statistical analysis was performed using spss version 23.0 software (SPSS Inc., Chicago, IL, USA) for Windows. *P* < 0.05 were considered statistically significant. Results from normally distributed variables were expressed as mean ± standard deviation (SD), and Student's *t*‐test or analysis of variance (ANOVA) test were performed to compare differences between groups. Non‐normal variables were expressed as medians and interquartile range (IQR), and differences between groups were compared by Mann–Whitney *U* or Kruskall–Wallis tests. The Kaplan–Meier test and odds ratio (OR) were performed to estimate renal survival and response to treatment in the presence of different histological findings. Multiple regression analysis was performed to assess independent factors associated by renal function outcome, defined by e‐GFR (end), slope‐eGFR and response to treatment, and binary logistic regression was used when outcome was defined by the 50% reduction in eGFR.

## Results

### Renal Function at Presentation and at Last Follow‐Up

The mean age of the 752 patients (male/female = 468/284) with PMN was 53.3 (15–85) years; they presented with serum creatinine (Screat) of 1.1 (0.5–9.6) mg/dl, eGFR (MDRD) of 69.3 (9.6–110) ml/min/1.73 m^2^ and urinary protein (Upr) of 7.3 (0.4–25) g/24 h. NS at presentation was evident in 622 (82.7%) and CKD in 162 (21.5%) patients. After a follow‐up of 122 ± 69 months, serum creatinine was increased to 1.4 (0.8–12.9) mg/dl, eGFR (MDRD) reduced to 58.6 (8.7–106) ml/min/1.73 m^2^ and Upr to 2.34 (0–12) g/24 h. Slope‐eGFR was estimated to −0.13 (−3.31 to 7.9) ml/min/1.73 m^2^/year for the whole cohort of patients, and 108 of 752 (14.3%) had >50% reduction of eGFR at the end of follow‐up. Complete response was evident in 371 of 752 (49.3%), partial in 205 of 752 (27.2%) and no response in 176 of 752 (23.4%).

### Histology

**Figure 1:**
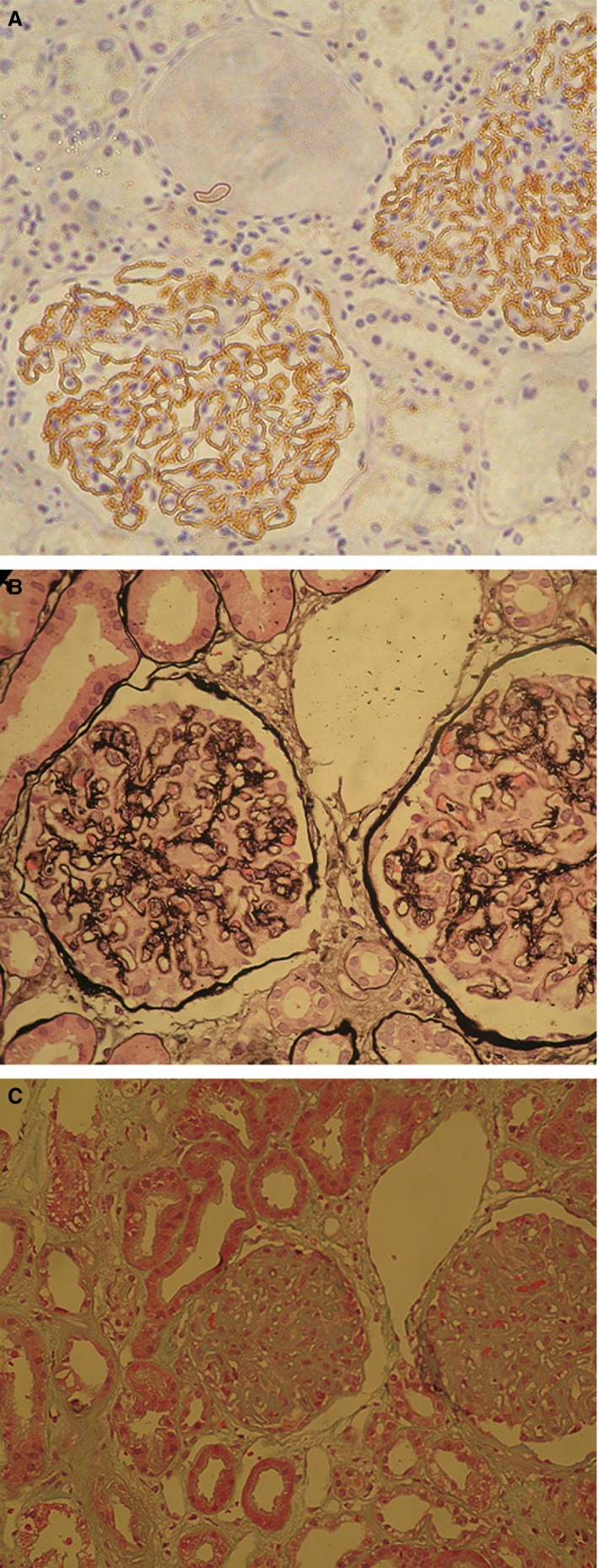
Granular deposits of C3 along glomerular membrane (**A**), presence of focal segmental sclerosis (FSGS) (**B**), tubuar atrophy grade 2 and interstitial fibrosis grade 3 (**C**) in primary membranous nephropathy (PMN).

Obsolescent glomeruli were present in 8.9 ± 13% of glomeruli, 243 of 752 (32%) of patients had FSGS and 384 of 752 (51%) had VH. TA was graded as stage 0 in 361 (48%), stage 1 in 310 (41.2%) and stage 2 in 81 (10.8%) of patients, while IF was rated as stages 0, 1 and 2 in 365 (48.6%), 325 (43.2%) and 62 (8.2%) of patients, respectively. IgG and C3 deposits were present in all patients on immunohistochemistry and/or immunofluoresence staining (Figure 1). FSTIV score was estimated as 0 in 176 (23.4%), 1 in 166 (22.1%), 2 in 180 (23.9%), 3 in 158 (21%) and 4 in 72 (9.6%) of the 752 patients.

### Parameters Correlated with Renal Function and Severity of Proteinuria at Presentation and at the End of Follow‐Up

Clinical parameters included age, male sex, presence of hypertension, degree of proteinuria, severity of dyslipidaemia, anaemia and histological findings: the degree of global sclerosis, presence of FSGS and VH and severity of TA and IF, and the FSTIV score had a positive correlation with renal function and some patients with severity of proteinuria at the time of diagnosis (Table 1).

**Table 1:** Correlation of renal function and degree of proteinuria at diagnosis with clinical and laboratory characteristics and histology findings

	eGFR at diagnosis	Uprot at diagnosis
	*P*	*P*
Age	<0.0001	NS
Sex	<0.0001	<0.0001
Hypertension	<0.0001	NS
Nephrotic syndrome	NS	<0.0001
Microscopic haematuria	NS	NS
Uprot (g/24 h)	0.01	–
STP (g/dl)	NS	<0.0001
Serum albumin (g/dl)	NS	<0.0001
Cholesterol mg/dl)	0.04	<0.0001
Triglycerides (mg/dl)	0.01	<0.0001
Hb (g/l)	<0.0001	NS
Histology		
Obsolescent glomeruli (%)	<0.0001	0.001
Focal sclerosis	0.004	0.05
Tubular atrophy	<0.0001	0.04
Interstitial fibrosis	<0.0001	<0.0001
Vessel hyalinosis	0.002	NS
FSTIV score	<0.0001	<0.0001

Uprot, Absolute proteinuria; NS, Not significant; STP, Serum total protein; Hb, Haemoglobin.

Patients with FSGS in renal biopsy had significantly impaired renal function and severe proteinuria at the time of diagnosis (mean rank = 267.76 and 235.98, *P = *0.02, and 260.22 and 288.12, *P* = 0.05, for eGFR and absolute proteinuria (Uprot) respectively, in FSGS (−) and FSGS (+) patients at the time of diagnosis. Differences in the outcome of renal function and rate of deterioration between FSGS (−) and FSGS (+) patients were also significant; mean rank = 235.98, 200.44, *P* = 0.004 for eGFR (end), 191.9 and 167.7, *P* = 0.04, for the slope‐eGFR and 252.10, 279.98, *P *< 0.0001 for Uprot (end), respectively.

**Figure 2:**
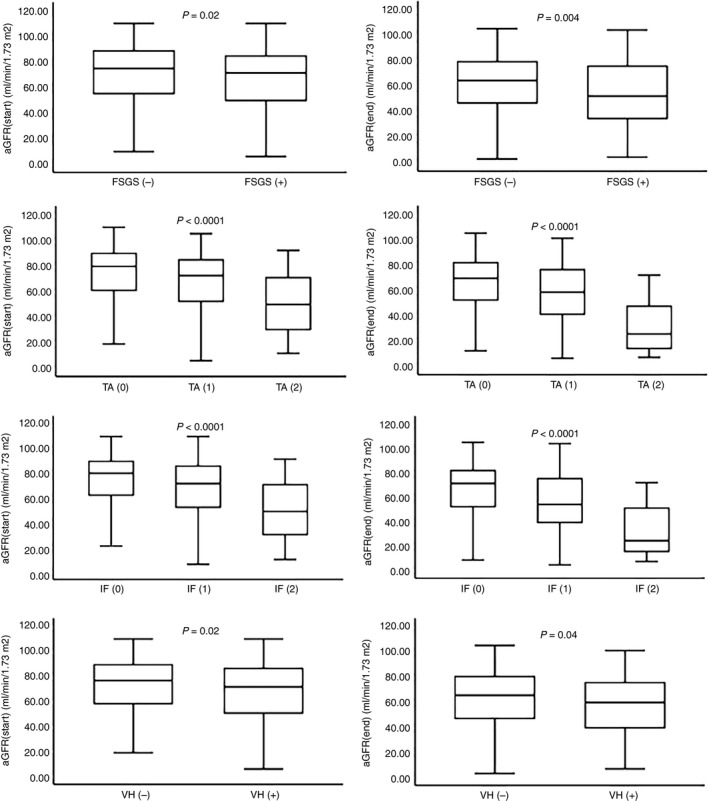
Differences in eGFR at diagnosis and at the end of follow‐up according to histology.

Similarly, the severity of TA and IF seemed to have a significant impact on eGFR at diagnosis; mean rank = 248.69, 214.43 and 131.13, *P* < 0.0001 for patients with TA rated as 0, 1, 2, respectively, and 283.72, 237.94 and 137.33, *P* < 0.0001, for IF estimated of 0, 1 and 2 degrees. Differences in Uprot showed a mean rank = 236.75, 269.27, 317.2, *P* < 0.002, for 0, 1, 2 IF, but no statistically significant difference for severity of TA. At the end of follow up, Kruskal–Wallis test applied for TA and IF showed a mean rank = 190.54, 156.90, 66.63, *P* < 0.0001 and 220.17, 170.75, 79.95, *P* < 0.0001, respectively, for eGFR (end), mean rank = 164.61, 159.6, 143.17, *P* = NS and 191.9, 180.5, 143.6, *P* = NS, respectively, for slope‐eGFR and finally, mean rank = 176.77, 178.96, 241.44, *P* = 0.002 and 194.02, 217.85, 298.89, *P* < 0.0001, respectively, for Uprot (end). The presence of vascular hyalinosis was also accompanied by a significantly reduced eGFR at the beginning and end of the study (mean rank = 262.41, 234.11, *P* = 0.02 and 197.05, 175.03, *P* = 0.04, respectively), while there were statistically significant differences in Uprot and slope‐eGFR. The impact of the four histological parameters in renal function at the beginning and end of the study is expressed in Figure 2 and their influence in response to treatment and the >50% reduction of eGFR in Table 2.

**Table 2:** The impact of histology on renal function outcome, estimated as response to treatment and >50% eGFR reduction, in the whole cohort of patients, in patients with nephrotic syndrome and patients with preserved renal function at diagnosis

	Response to treatment			>50% reduction in eGFR and/or ESRD		
	OR	95% CI	*P*	OR	95% CI	*P*
All patients						
FSGS	1.792	1.157–2.778	0.009	3.259	1.779–5.970	<0.0001
TA	2.272	1.612–3.202	<0.0001	3.113	1.916–5.059	<0.0001
IF	2.360	1.673–3.33	<0.0001	2.760	1.716–4.439	<0.0001
VH	1.615	1.038–2.513	0.033	1.331	0.730–2.426	NS
FSTIV	1.569	1.317–1.869	<0.0001	1.783	1.39–2.289	<0.0001
Patients presenting with nephrotic syndrome						
FSGS	1.771	1.091–2.875	0.02	3.414	1.793–6.503	<0.0001
TA	2.622	1.78–3.862	<0.0001	2.722	1.633–4.537	<0.0001
IF	2.383	1.633–3.479	<0.0001	2.323	1.416–3.810	0.001
VH	1.742	1.072–2.829	0.025	1.232	0.655–2.317	NS
FSTIV	1.550	1.282–1.873	<0.0001	1.64	1.271–2.116	<0.0001
Patients presenting with eGFR ≥ 60 ml/min/1.73 m^2^						
FSGS	1.766	1.009–3.09	0.04	3.402	1.714–6.75	<0.0001
TA	2.308	1.439–3.7	0.001	4.057	2.165–7.602	<0.0001
IF	2.386	1.506–3.779	<0.0001	3.279	1.827–5.885	<0.0001
VH	1.382	0.8–2.388	NS	1.459	0.746–2.853	NS
FSTIV	1.516	1.218–1.888	<0.0001	1.829	1.385–2.415	<0.0001

ESRD, end‐stage renal disease; FSGS, Focal segmental sclerosis; TA, Tubular atrophy; IF, Interstitial fibrosis; VH, Vascular hyalinosis; OR, Odds ratio; CI, Confidence interval.

Multiple regression analysis showed that the independent factors correlated with renal function at diagnosis were age (*r* = −0.5, *P* < 0.0001), degree of proteinuria (*r* = 0.43, *P* < 0.0001) and the severity of TA (*r* = 0.4, *P *< 0.0001).

Similarly, multiple regression analysis performed to estimate independent factors correlated with the outcome of renal function, eGFR (end), response to treatment and >50% reduction in eGFR and rate of declining eGFR eGFR (slope‐eGFR). Significant independent parameters correlated with (end) were eGFR (start) (*r* = 0.6, *P* < 0.0001), age (*r* = −0.6, *P* < 0.0001), FSGS (*r* = −0.6, *P* < 0.0001) and TA (*r* = −0.6, *P* < 0.0001). Independent factors correlated with response to treatment were the degree of IF (*r* = 0.3 and *P* < 0.0001) and presence of FSGS (*r* = 0.4, *P* < 0.0001). Binary logistic analysis proved that age (*P* = 0.003), FSGS (*P* = 0.001) and TA (*P* = 0.02) were independent parameters correlated with the endpoint of >50% eGFR reduction. Slope‐eGFR was associated with eGFR (start) (*r* = −0.4, *P* < 0.0001), age (*r* = −0.5, *P* < 0.0001) and TA (*r* = −0.5, *P* < 0.0001) in multiple regression analysis.

### The Influence of Histology in Patients with NS and in Patients with Preserved Renal Function

From the whole cohort of 752 patients, 622 (82.7%) had NS and 590 (78.5%) had preserved renal function (eGFR ≥ 60 ml/min/1.73 m^2^) at diagnosis.

The presence of FSGS and VH had a similar distribution, and the degree of TA and IF were also comparable between patients who presented with or without NS. Patients with preserved renal function had a significantly reduced frequency of FSGS (χ^2 ^= 4.8, *P* = 0.02) and VH (χ^2^ = 10.51, *P* = 0.001) and lower degrees of TA and IF (χ^2^ = 47.38, *P* < 0.0001, and χ^2^ = 54.63, *P* < 0.0001, respectively) (Table 3).

**Table 3:** Differences in the presence and severity of histological findings between patients presenting without or with CKD

eGFR	≥60 ml/min/1.73 m^2^	<60 ml/min/1.73 m^2^	χ^2^	*P*
*n*	590	162		
FSGS (−)	411	98		
FSGS (+)	179	64	4.8	0.02
VH (−)	307	61		
VH (+)	283	101	10.51	0.001
TA 1	304	52		
TA 2	252	74		
TA 3	34	36	47.38	<0.0001
IF 1	314	51		
IF 2	250	78		
IF 3	26	33	54.63	<0.0001

CKD, Chronic kidney disease; FSGS, Focal segmental sclerosis; TA, Tubular atrophy; IF, Interstitial fibrosis; VH, Vascular hyalinosis.

**Figure 3:**
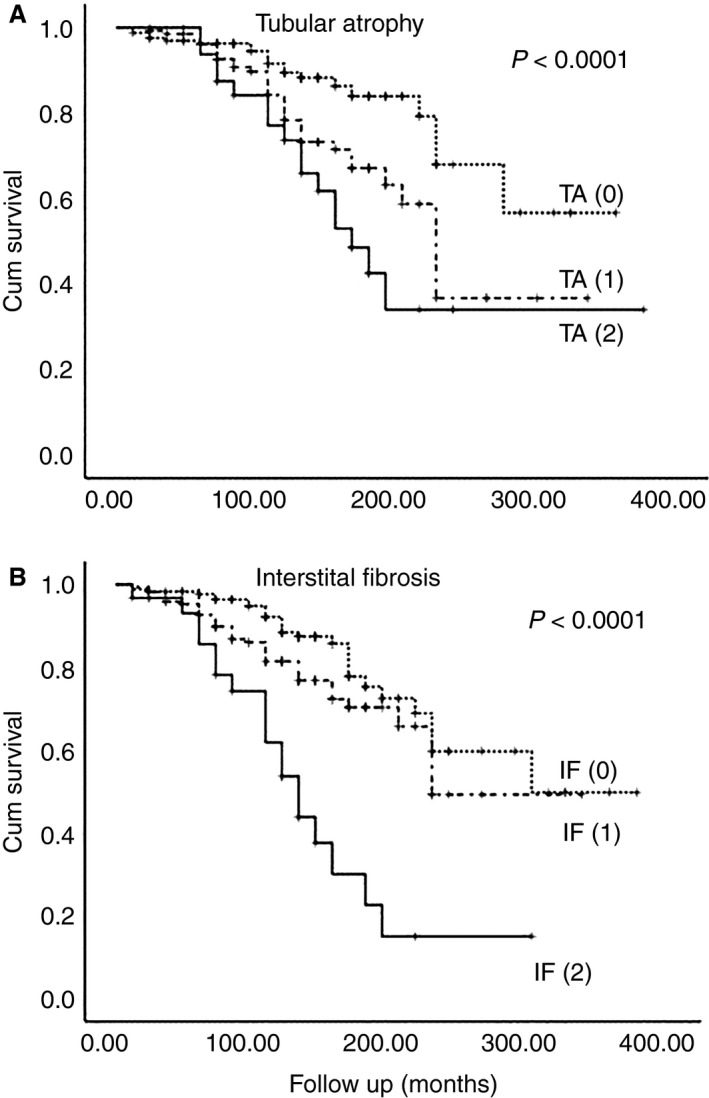
Impact of tubular atrophy (**A**) and interstitial fibrosis (**B**) in the response to treatment in the presence of nephrotic syndrome (NS).

Response to treatment in patients who presented with NS was correlated with all four histological parameters: the presence of FSGS (*P* = 0.005) and VH (*P* = 0.006) and the degree of TA (*P* < 0.0001) and IF (*P* < 0.0001) (Figure 3). However, slope‐eGFR was associated only with the presence of FSGS in renal biopsy, and was estimated to −2.2 ± 4.3 versus −1.2 ± 3.2 ml/min/1.73 m^2^/year, *P* = 0.02, in FSGS (+) and FSGS (−), respectively.

In patients with eGFR ≥ 60 ml/min/1.73 m^2^, response to treatment was correlated with the presence of FSGS and the degree of TA and IF (*P* = 0.01, *P* = 0.02, *P* < 0.0001, respectively). The same findings had a significant impact in the rate of renal function deterioration, as indicated by the slope‐eGFR, −2.9 ± 3.8 versus −1.2 ± 3.7 ml/min/1.73 m^2^/year, *P* = 0.01, in FSGS (+) and FSGS (−), respectively, −1.8 ± 3.9, −1.8 ± 3 and −4.2 ± 3.3 ml/min/1.73 m^2^/year, *P* = 0.05, for patients with TA estimated as 0, 1 and 2, respectively, and −1.8 ± 4, −2.3 ± 3.4 and −4.7 ± 3.3 ml/min/1.73 m^2^/year, *P* = 0.05, for patients with IF estimated as 0, 1 and 2, respectively.

The ORs of the four histological parameters, performed initially on the whole cohort of patients and subsequently to patients with NS and patients with preserved renal function for the response to treatment and deterioration of renal function, defined as >50% reduction of eGFR and/or ESRD, are shown in Table 2.

### The Influence of Histology in the Effect of Immunosuppressive Treatment

**Figure 4:**
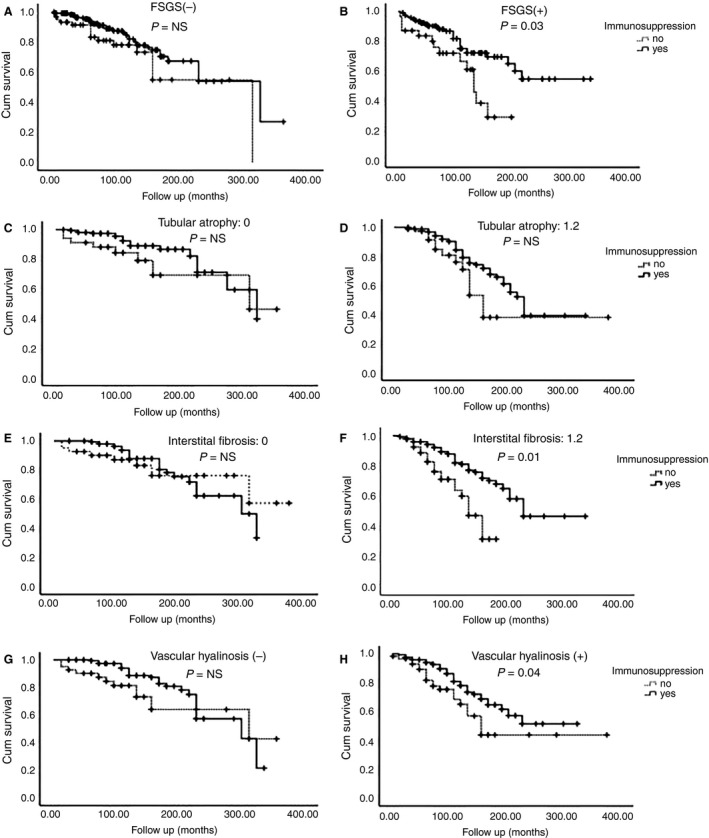
The result of immunosuppressive treatment in the absence (**A**,**C**,**E**,**G**) or presence (**B**,**D**,**F**,**H**) of focal segmental sclerosis (FSGS), tubular atrophy (TA), interstitial fibrosis (IF) and vascular hyalinosis (VH), respectively.

**Figure 5:**
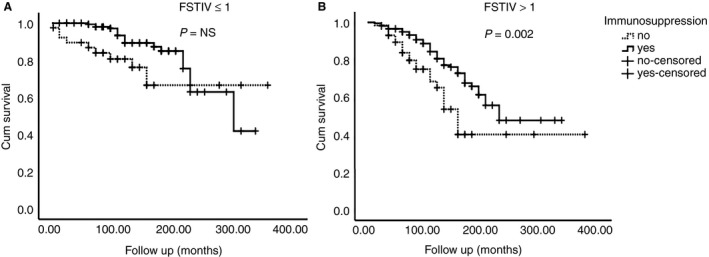
The result of immunosuppressive treatment in patients with low (≤1) (**A**) or high FSTIV score (>1) (**B**).

We evaluated the impact of FSGS, TA, IF and VH in the response to immunosuppression. Immunosuppressive treatment had a beneficial effect in patients with FSGS, IF (grades 1–2) and VH on renal biopsy, but not in those without FSGS, IF and VH (Figure 4). Moreover, the combination FSTIV score also seemed to positively influence effect of immunosuppressive treatment. In order to simplify analysis, we divided patients into those with FSTIV score to ≤1 (*n* = 342) and with FSTIV score >1 (*n* = 410), and evaluated the possible beneficial effects from immunosuppressive treatment. As depicted in Figure 5, immunosuppressive treatment had no effect in the former group; besides, there was a significantly better outcome of renal function after immunosuppression in the second cohort of patients with advanced histology, and this effect was constant even after long‐term follow‐up.

## Discussion

Diagnosis of PMN and exclusion of secondary cases are widely based on immunohistochemistry and/or immunofluorescent findings, and classification currently depends on electron microscopy. However, electron microscopy is not widely available in every centre, and current classification has not been proved to correlate with disease outcome.^14^, ^16^ Therefore, in the present study we intended to search for a simplified grading in PMN, including histological parameters, which can be easily identified and evaluated in a routine base optical microscopy.

Histology in MN does not include proliferative lesions. Mesangial proliferation is unusual and may act as an indicator of secondary forms of disease, and crescent formation and endothelial proliferation are extremely rare. Besides, tubulointerstitial lesions are widely accepted as the most important findings for disease outcome.^17^, ^18^, ^19^ We therefore decided to integrate only four parameters in the assessment of renal biopsies, namely: (i) the presence of FSGS, representing glomerular damage, (ii) degree of TA, (iii) severity of IF, to assess tubulointerstitial damage and (iv) presence of VH, to estimate the vasculature implication. Our objective was to review the importance of ordinary histological findings into a routine pathology assessment of PMN, and to provide parameters with prognostic significance and indices to guide treatment approach. Furthermore, we combined the above four histological parameters in a novel, concise score (the FSTIV score), and assessed its significance in predicting response to immunosuppressive treatment and renal function outcome in long‐term follow‐up.

FSGS and vascular hyalinosis were present in one‐third and half of cases, respectively, while moderate to severe (grades 1 and 2) tubular atrophy and interstitial fibrosis were seen in more than 50% of the patients. Clinical presentation of our patients covered all aspects of disease, from mild proteinuria and normal renal function to severe renal impairment with or without nephrotic syndrome, hypertension and haematuria. Clinical parameters, such as age, male sex, hypertension and degree of proteinuria and histological findings, including obsolescent glomeruli, presence of focal sclerosis and vascular hyalinosis, severity of tubular atrophy and interstitial fibrosis, correlated with severity of renal function impairment at the time of renal biopsy. These findings are in agreement with previous results, which showed that clinical parameters with predictive significance were age, male sex, hypertension and renal insufficiency.^20^

There has been a great deal of debate concerning the accuracy and efficacy of the levels of proteinuria and eGFR in determining treatment strategies, but although the story goes back to the early 1980s there is still no accurate answer, even after the use of biomarkers such as beta‐2‐microglobulin or N‐acetyl‐beta‐glucosaminidase, or the description of anti‐PLA2R antibodies.^20^, ^21^, ^22^, ^23^, ^24^ Therefore, histology should be thoroughly analysed in order to estimate the severity of disease and make decisions concerning treatment choices. In the present study we found that, in the whole cohort of patients, all four parameters examined, including FSGS, TA, IF and VH, had a significant negative correlation with eGFR levels at presentation and also with renal function outcome; however, the most important of them, as proved by the multiple regression analysis, being the presence of FSGS and severity of TA and IF.

In the present study, we estimated eGFR at the end of follow‐up and the ≥ 50% reduction of eGFR in order to evaluate outcome of the disease, and we also estimated slope‐eGFR as an indicator of renal function deterioration rate. We also used KDIGO definition to assess the response to treatment approach. KDIGO is the most important and widely accepted clinical practice guideline document for the assessment and treatment of primary and secondary glomerular diseases. KDIGO guidelines for PMN are mainly based upon two main clinical characteristics, the presence of NS and the presence of CKD. All treatment strategies and decisions, and the decision to start on immunosuppressive treatment, are made upon the degree of proteinuria and the preservation of renal function.^15^ Therefore, additionally to the whole cohort of patients, we examined the importance of histology in the presence of NS and preserved renal function.

Regarding patients who presented with NS; FSGS, TA and IF had a significant role in final outcome. However, the presence of FSGS was the only finding associated with the rate of declining renal function. Among patients with preserved renal function, FSGS, TA and IF were important in both outcome, and rate of deterioration.

The evaluation of FSGS as a predictor factor of renal function outcome has gained much interest but results remain conflicting, with some investigators supporting a pathogenic role of FSGS in declining renal function, providing FSGS and the severity of tubulointerstitial fibrosis as the only independent factors of disease outcome,^25^, ^26^, ^27^, ^28^ while others doubt that there is any correlation of FSGS with renal function outcome.^29^, ^30^

Based on the results from our study, FSGS and VH are important findings in the presence of NS, and additionally the presence of FSGS predicts a worse outcome in patients with preserved renal function at diagnosis. Apparently, neither FSGS nor VH should be regarded as findings secondary to co‐factors, such as advanced age and hypertension, but they seem to act independently as histological parameters determining progression of renal function.

The simplified FSTIV score, resulting from the combination of the most common histological findings in PMN, also had a significant impact on the outcome and response to treatment. A similar score, the MEST score, based on the presence of mesangial (M), endocapillary (E), hypercellularity, glomerular sclerosis (S) and tubular atrophy (T), has been previously applied to estimate the severity of IgA nephropathy.^31^, ^32^ FSTIV score is based on optical microscopy findings, can easily be described by a pathologist, and can assist the clinician to estimate disease severity, predict renal function outcome and decide upon treatment approach.

An interesting finding of our study was the influence of histology on the effect of immunosuppressive treatment. Regardless of clinical presentation, patients with FSGS, VH and IF (degrees 1 and 2) in renal biopsy had a significantly better outcome when they received immunosuppressive treatment combined with those who did not. Furthermore, patients with an FSTIV score >1 seemed to benefit from immunosuppression, instead of those with less severe histology, and this beneficial effect was stable after long‐term follow‐up. These findings, in agreement with previous results which showed that even patients with severe tubulointerstitial injury may gain remission after treatment,^33^ support the administration of immunosuppressive treatment in PMN, especially in the presence of the above histological features.

In conclusion, after evaluation of certain histological findings in PMN, we found that FSGS, TA, IF and VH are significant parameters predicting renal function outcome and response to treatment and should be considered as imperative factors in the management of PMN. The combination of these four findings revealed that the FSTIV score, a novel, simplified and comprehensive score, can not only help in grading PMN, but can also be used as guidance for treatment strategies.
